# Selection for Growth Performance in Broiler Chickens Associates with Less Diet Flexibility

**DOI:** 10.1371/journal.pone.0127819

**Published:** 2015-06-04

**Authors:** Jana Pauwels, Frank Coopman, An Cools, Joris Michiels, Dirk Fremaut, Stefaan De Smet, Geert P. J. Janssens

**Affiliations:** 1 Laboratory of Animal Nutrition, Faculty of Veterinary Medicine, Ghent University, Merelbeke, Belgium; 2 Department of Applied Biosciences, Ghent University, Gent, Belgium; 3 Laboratory for Animal Nutrition and Animal Product Quality, Department of Animal Production, Ghent University, Melle, Belgium; Hospital for Sick Children, CANADA

## Abstract

Global competition for high standard feed-food resources between man and livestock, such as industrial broilers, is a concerning problem. In addition, the low productivity of scavenger chickens in developing countries leaves much to be desired. Changing the ingredients, and therefore, the nutrient composition of feed intake by commercial fed as well as scavenger chickens seems like an obvious solution. In this study, the ability of four broiler chicken breeds to perform on a commercial versus a scavenger diet was tested. The four broiler breeds differed genetically in growth potential. A significant (*P* < 0.01) negative effect of the scavenger diet on the bodyweight of the fast growing breeds was found and this effect decreased with decreasing growth rate in the other breeds. These differences in bodyweight gain could not be explained by differences in nutrient digestibility but were caused by the lack of ability of the fast growing breeds to increase their feed intake sufficiently.

## Introduction

The feeding of industrial broiler chickens is often criticized because of the extensive use of feed sources which are neither socially nor ecologically sustainable [1–4]. The diet of intensively-raised broilers consists mainly of maize, soy and wheat [5–7], ingredients that could also be used directly in the human diet [8]. Ravindran [9] and Farrell [10] proposed alternative ingredients, less sought after in the human diet, that could be used in the chickens’ diet.

The low productivity of scavenger chickens in developing countries is often blamed on the lack of concentrated diets [10,11]. The diet of scavenger chickens contain, for example, crude fiber levels up to more than 100g/kg dry matter [12]. While Jørgensen *et al*. [13] reported that broilers (by fermentation of three different non-starch polysaccharide (**NSP**) rich substrates: pea fibre, wheat bran and oat bran) extract a maximum of 42kJ/d, representing only 3 to 4% of the daily metabolisable energy intake, hereby confirming the widely accepted idea about the low energetic value of NSP-rich diets for poultry [14].

Solving the problems for both the industrial and the rural chickens is especially interesting since chickens are a widespread food source around the globe. The United Nation’s Food and Agriculture Organisation (FAO) estimated that there were nearly 22 billion living chickens in 2012 [15]. This is the equivalent of more than three chickens per person. Moreover, in developing countries, chickens are often the main source of animal protein through their meat and eggs and most of these chickens stem from indigenous, slow growing, breeds [16,17].

In this study four different chicken breeds, with growth rates between 30 and 60g/d, were used to plot a range in growth rate between the slow growing scavenger chicken on one side and the fast growing industrial broiler chicken. Two diets were used to feed these chickens, one was a commercial industrial diet while the other was based on reported scavenger diets [12,18–20]. The average daily gain, average daily feed intake, feed conversion ratio and the length of the *tarsometatarsus* were registered for all breeds on each of the diets. The aim of this study was to monitor the effect of the scavenger diet in relation to breed-specific growth rate.

## Materials and Methods

### Animals and housing

The experiment was carried out according to the guidelines of the Ethics Committee of Ghent University (Belgium) for the humane care and use of animals in research. According to the Decree of May 29th 2013, no explicit approval of the Ethical Committee is needed. The animals are only fed two different diets (none of both is toxic) and no invasive procedures are applied to the chickens. National regulations allowed to run this type of trial without particular per case agreement by the Ethical Committee, as long as no pain or suffering is induced during the trial, as is the case here. From each pen, one animal was sacrificed (injection IV of 1ml sodium pentobarbital, Release) in order to collect data for other researchers working on a different research topic in the idea of reducing the number of experimental animals. Again, if no pain or suffering was induced during the trial, regulations allowed to euthanize experimental birds preceding sampling, hence adhering to Belgian regulations on animal welfare. The researcher in charge was a veterinarian who completed the course on “Laboratory Animal Science” to obtain the degree C within the FELASA regulations, and is therefore authorised to handle and sacrifice animals. The complete trial took place at Ghent University (site Melle). After the end of the experiment, the remaining chickens were given to and taken home by the students who helped during the trial.

Four different broiler breeds were selected based on their commercial growth rate from hatching weight to slaughter weight (hereafter referred to as breed-specific growth rate): Cobb 500 (60 g/d) [21], Cobb-Sasso 175 (46g/d) [22], Sasso (XL44 × SA₅₁(A)) (38g/d) [23] and Sussex (Sussex × SA₅₁(A)) (30g/d) [24]. A total of 240 one day old male chicks, sixty from each breed, were obtained from the same hatchery (‘t Gulden Ei, Kruishoutem, Belgium). From each breed, 15 chicks were randomly assigned to 4 pens littered with wood shavings. Each pen contained 15 animals and breeds were not mixed. During the first week, 23 hours of light were provided per day and from day 8 the light period was reduced to 18 hours per day. At hatching all chicks were vaccinated against Newcastle disease (**NCD**) (Nobilis, NDC2), infectious bronchitis (Nobilis, IB MA5) and Marek’s disease (Fort Dodge, Poulvac Marek HVT). Four day old chicks were vaccinated against coccidiosis (Intervet, Paracox-5). At 19 days the NCD (Nobilis, ND Clone 30) vaccination was repeated and a vaccination against Gumboro (Nobilis, Gumboro D78) disease was performed at 22 and 25 days.

### Diets and Treatments

From hatching day (day 0), two pens of each breed were fed the commercial diet and two pens from each breed were fed the scavenger diet. A representative sample from eight randomly chosen feed bags of each diet was taken and pooled for both diets. Both samples were analysed in duplicate for nutritional values Table 1. The commercial diet (Fini Pur Croc, Versele-Laga, Deinze, Belgium) was mainly composed of wheat and soybean meal and it contained two enzymes: 6-phytase and E1617-Endo-1,4-β-xylanase. This diet was ground into flour and 10 g/kg of Celite (VWR International, Leuven, Belgium) was added as source of acid insoluble ash (**AIA**) in order to determine diet digestibility [25]. The second diet was based on reported scavenger diets [12,18–20] and contained 930 g/kg Austru 2 Growth (Versele-Laga, Deinze, Belgium), 40g/kg dried mealworms (*Tenebrio molitor*), 20g/kg dried lucerne and 10g/kg Celite. All components were ground into flour and mixed until a homogenous feed was acquired. The Austru 2 Growth was mainly composed of wheat, sunflower meal and corn and contained no additional enzymes. To accustom the animals to the scavenger diet, groups fed this diet received a mixture of one third scavenger diet and two thirds commercial diet from day 0 to 5, two thirds scavenger diet and one third commercial diet from day 6 to 10, and from day 11 onwards only the scavenger diet was offered. Drinking water was provided ad libitum in drinking cups.

**Table 1:** Nutrient and energy concentration of both test diets: commercial and scavenger diet.

	*Commercial diet*	*Scavenger diet*
*Dry matter (g/kg)*	902	911
*Crude protein (g/kg)*	215	187
*Ether extract (g/kg)*	81	47
*Crude fibre (g/kg)*	40	143
*Acid detergent fibre (g/kg)*	13	18
*Neutral detergent fibre (g/kg)*	67	68
*Ash (g/kg)*	62	79
*Acid-insoluble ash (g/kg)*	15	15
*Metabolisable energy (MJ/kg)*	15	10

### Measurements and sampling

Starting from day 0, the chickens were weighed weekly until day 36 and the average bodyweight per pen was calculated. Average daily gain (**ADG**), average daily feed intake (**ADFI**) and feed conversion ratio (**FCR**) were calculated over the period of 36 days and were corrected for bodyweight at hatching and mortality during the trial. To collect excreta for the digestibility trials, a container (1cm × 40cm × 1m) with a grid was placed in each pen weekly, from the second week on. After four hours the excreta were collected from the container and stored in a freezer (-20°C). On day 36, the length of the *tarsometatarsus* of each bird was measured by flexing the leg and registering the distance between the medial condyle of the *fibula* and the *trochlea* for *metatarsus III*. The average was calculated per pen and the ratio of bodyweight to *tarsometatarsus* length was calculated as in Deeb and Lamont [26]. Each week the consistency of the litter was observed by the same person.

### Analysis

Both diets were analysed for dry matter (**DM**), ash, acid insoluble ash (**AIA**), crude fat (**EE**), crude fiber (**CF**), neutral detergent fiber (**NDF**), acid detergent fiber (**ADF**) and crude protein (**CP**) Table 1. The DM and ash content were determined by drying the feed to a constant weight at 103°C and combustion at 550°C, respectively. The AIA content was determined using the procedure of Van Keulen and Young [27], as adapted by Atkinson *et al*. [28]. The diethyl ether extract was analyzed with the Soxhlet method (ISO, 1973). Crude fiber was determined using the Association of Official Analytical methods (Method 962.09 and 985.29, 1995). To determine NDF and ADF, the methods of Van Soest *et al*. [29] were used. The Kjeldahl method (ISO 5983–1, 2005) was used to determine CP (6.25 x N). Excreta were freeze-dried at -50°C (Coolsafe, Labogene, Denmark) and homogenized. In the excreta, EE, CF, Ash and AIA were analysed as described above. The CP content (6.25 x N) (Kjeldahl method (ISO 5983–1,2005)) in the excreta of birds needed correction for uric acid (**UA**) as birds excrete faeces and urine together [30] (Eq (1)). This was performed spectrophotometrically according to Terpstra and de Hart [31]. The external marker method with AIA as external marker was used to calculate apparent fecal digestibility as performed by Sales and Janssens [32,33] (Eq (2), AFDₓ, with x = EE, CF and CP). Based on the EE, CF and ash percentage of DM, ME was calculated according to Wiseman [34] (Eq (3)).

### Calculations

$$CP=(N_{total}-N_{UA})\times 6.25$$ (1)

$$AFD_{x}(\%)=100-100\times \frac{X_{excreta}\times AIA_{feed}}{AIA_{excreta}\times X_{feed}}$$ (2)

ME(MJ)=(3951+54.4×EE−88.7×CF−40.8×Ash)×0.92×0.004184 (3)

### Statistical analysis

Statistical analyses were performed using RStudio (Version 0.98.507, RStudio Inc, 2009) and statistical significance was set at *P* < 0.05. For all analyses pens were considered as the experimental unit and non-parametric statistics was performed for data analysis as normality of data could not be verified in the current trial set up. Both weekly bird weight (week 0 to 5) and digestibility of nutrients (week 2 to 5) were subjected to longitudinal non parametric analysis using the f2.ld.f1() function of the nparLD package with diet and breed as the whole-plot factors and time as the sub-plot factor. Decisions on significance were made based on the ANOVA type test statistics provided by the latter function [35]. To identify the effect of time, a pairwise comparison was done between the different time points by means of the mctp.rm() function of the nparcomp package. To estimate the effect of breed-specific growth rate and diet on average daily feed intake (**ADFI**), average daily gain (**ADG**), feed conversion ratio (**FCR**) and the *tarsometatarsus* length a linear permutation regression with 5000 replicates (lmp() of the lmPerm package) was performed. Each of the 16 pens was randomly assigned to one of the two diets. To differentiate the effect of diet and of breed-specific growth rate, the null hypothesis of no effect was used. The alternative hypothesis is that there is an effect of diet and breed-specific growth rate on the parameters [36,37]. The maximum number of performed permutations was set at 5000 because of a total of 16! possible permutations for this data set. To exclude the possible error by only taking 5000 permutations the test was performed a thousand times with each time another set of 5000 random permutations. From these thousand permutation tests the maximum *P*-value was selected and represented together with the corresponding estimates of the linear regression which is presented as:

$$y=\mu +\alpha_{i}+\beta_{j}+\alpha \beta_{ij}+\varepsilon_{ij}$$

(with μ = intercept, α = breed-specific growth rate, β = diet, αβ = interaction growth rate × diet and ε = the random error term). The commercial diet was considered as the reference in this regression. Statistical significance was set at *P* < 0.05 and results were reported as mean ± standard deviation.

## Results

### Growth performance

**Fig 1:**
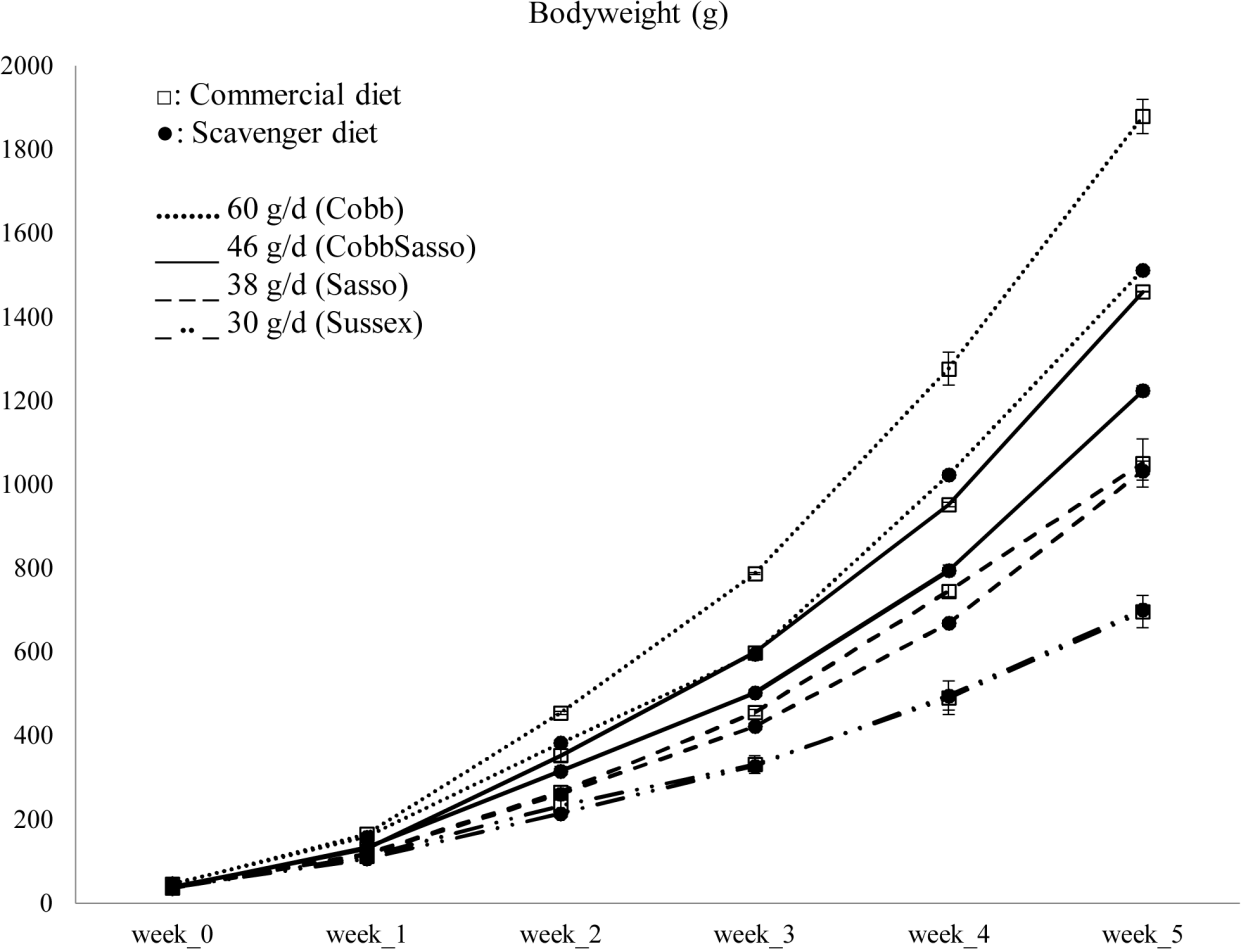
Bodyweight (± standard deviation) of four chicken breeds, each with its breed-specific growth rate, fed either a commercial or a scavenger diet.

A higher bodyweight was found with increasing time, increasing breed-specific growth rate and for consumption of the commercial diet (Fig 1). For both diets, Cobb chickens achieved the highest bodyweight, each week. Sussex chickens always had the lowest bodyweight compared to the breeds with a higher breed-specific growth rate, but there was no significant effect resulting from diet on the bodyweight of Sussex chickens. Longitudinal analysis indicated a significant interaction of the factors breed, diet and time (*P* < 0.001) on the bodyweight.

**Fig 2:**
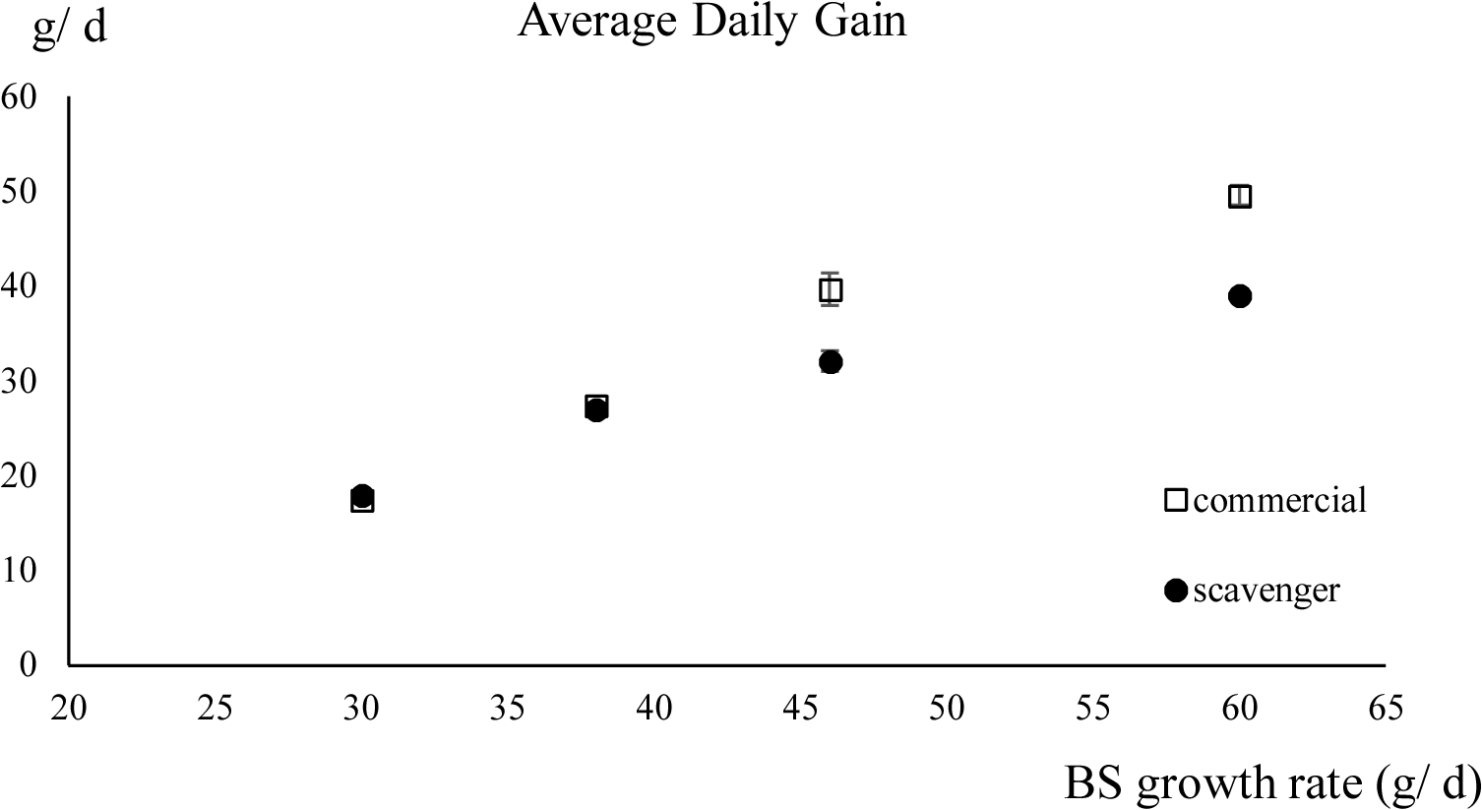
The average daily gain (± standard deviation) of chickens fed the scavenger or the commercial diet in relation to their breed-specific (BS) growth rates. BS growth rates: 60g/ d (Cobb), 46g/ d (CobbSasso), 38g/ d (Sasso) and 30g/ d (Sussex).

The linear regression of ADG was determined by breed-specific growth rate (*P* < 0.001), diet (*P* < 0.01) and the interaction of both factors (*P* < 0.01) Table 2 (Fig 2). The coefficient for the diet × breed-specific growth rate interaction was -0.39. This means that, compared to a commercial diet, the ADG of the chickens on a scavenger diet decreased with 0.39g/d when the growth rate increased with 1g/d.

**Table 2:** The linear regression between both factors (BS growth rate and diet) and ADG, ADFI, FCR, TMT and BW/ TMT.

	Intercept	BS growth rate	Diet	BS growth rate × Diet	*R^2^*	*P*
ADG	30.4 ***	0.85 ***	-4.4 **	-0.39 **	0.97	***
ADFI	71.3***	1.3 ***	14.5***	-0.33	0.97	***
FCR	2.5 ***	-0.03 ***	0.81 ***	-0.01	0.88	***
TMT	8.3 ***	0.05 ***	-0.56 **	-0.03	0.82	***
BW/*TMT*	140.5 ***	3.1 ***	-8.7	-1.0	0.91	***

*Coefficients of the linear regression of broiler chickens differing in breed-specific growth rate fed a commercial versus a scavenger diet correlated to the average daily gain (ADG)*, *average daily feed intake (ADFI)*, *feed conversion ratio (FCR)*, *length of tarsometatarsus (TMT) and the ratio between the bodyweight and the length of the tarsometatarsus (BW/TMT)*. *The factors in the equation are breed-specific (BS) growth rate and diet*. *The commercial diet was considered as the reference*. *The BS growth rates for each of the breeds used in this experiment are*: *60g/ d for Cobb*, *46g/ d for CobbSasso*, *38g/ d for Sasso and 30g/ d for Sussex*. *R^2^ and the P-value of the linear regression models are given in the right colomns*.

*Superscripts represent the P-value*:

* = <0.*05*

** = <0.*01*

*** = <0.*001*.

### Feed intake

**Fig 3:**
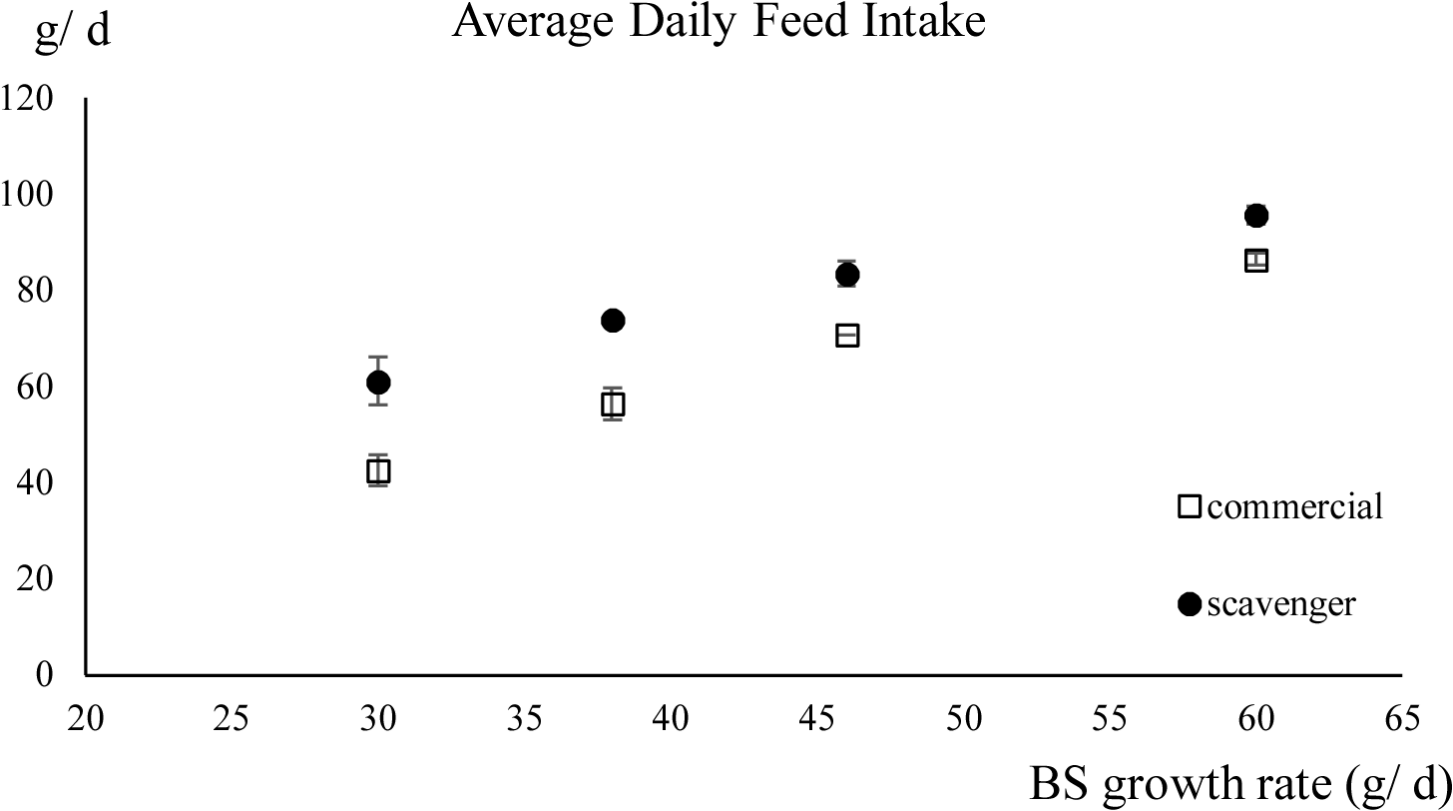
The average daily feed intake (± standard deviation) of chickens fed the scavenger or the commercial diet in function of their breed-specific (BS) growth rates. BS growth rates: 60g/ d (Cobb), 46g/ d (CobbSasso), 38g/ d (Sasso) and 30g/ d (Sussex).

The ADFI was significantly higher for the scavenger diet (*P* < 0.001) and with increasing breed-specific growth rate (*P* < 0.001). No significant interaction between breed-specific growth rate and diet on the ADFI was found (*P* > 0.05) Table 2 (Fig 3).

### Feed conversion

**Fig 4:**
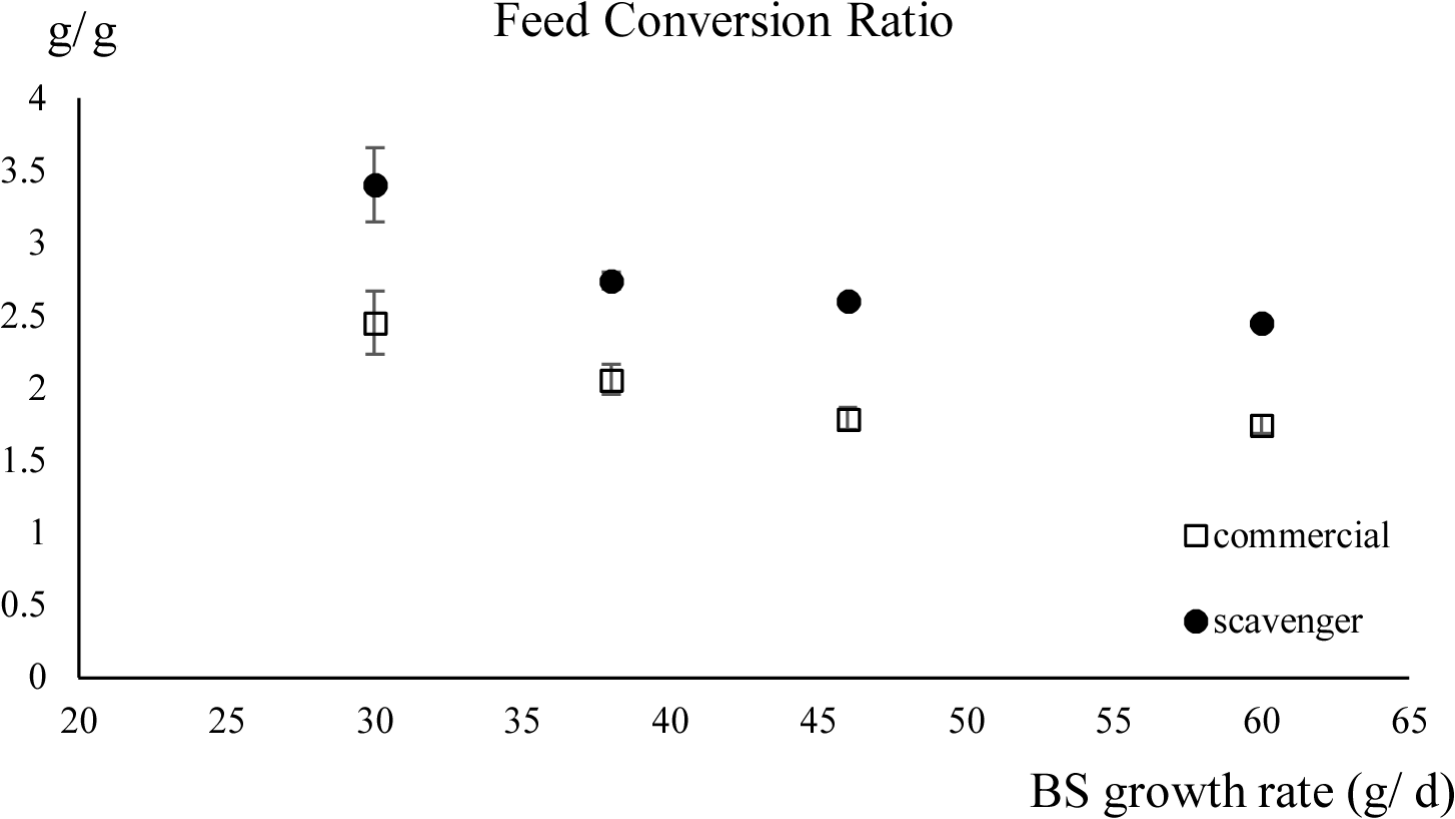
The feed conversion ratio (± standard deviation) of chickens fed the scavenger or the commercial diet in function of their breed-specific (BS) growth rates. BS growth rates: 60g/ d (Cobb), 46g/ d (CobbSasso), 38g/ d (Sasso) and 30g/ d (Sussex).

No significant interaction between breed-specific growth rate and diet on the FCR was found (*P* = 1). The scavenger diet significantly increased the FCR (*P* < 0.001) and a higher growth rate decreased the FCR (*P* < 0.001) Table 2 (Fig 4).

### *Tarsometatarsus* length

**Fig 5:**
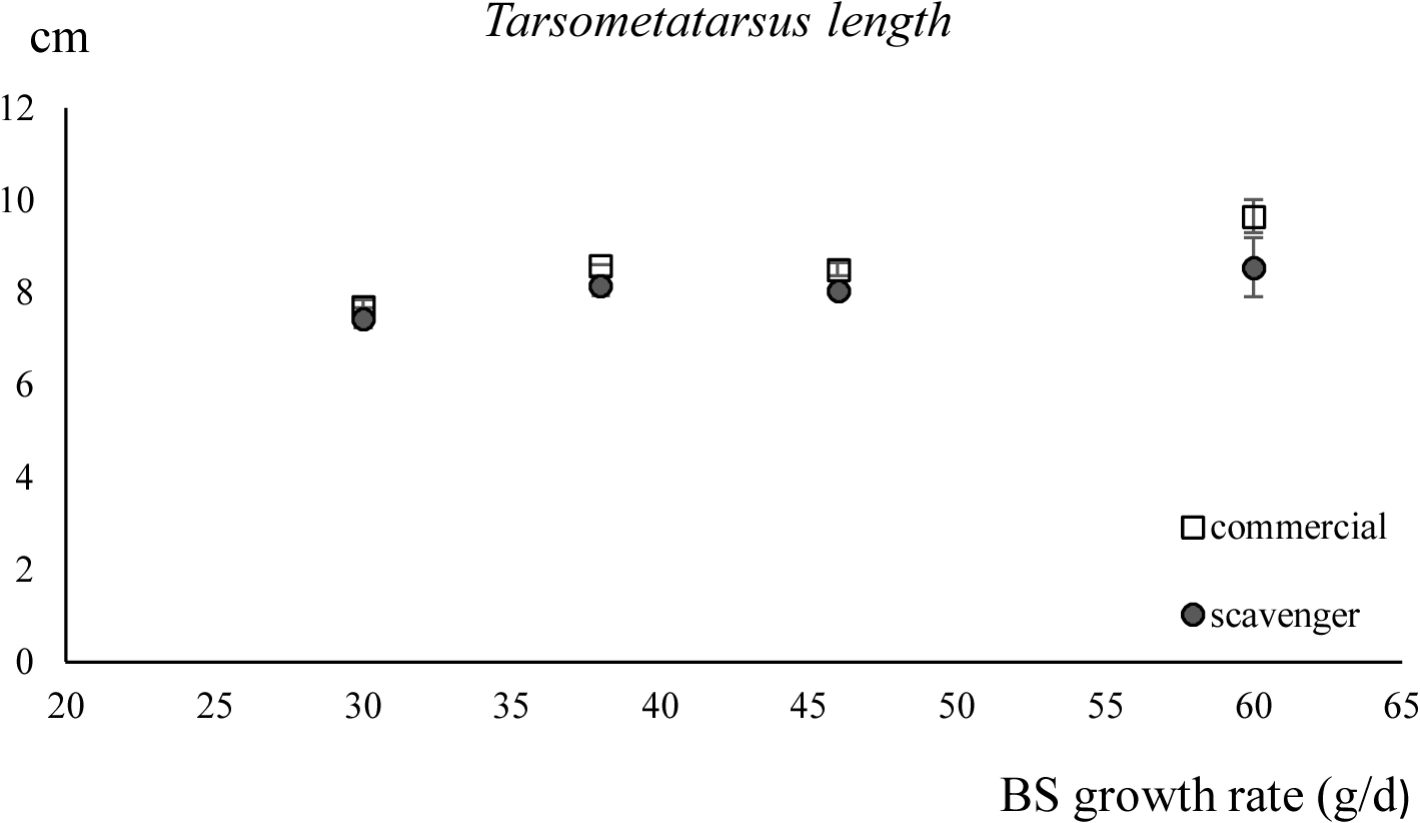
The length of the *tarsometatarsus* (± standard deviation) of chickens fed the scavenger or the commercial diet in function of their breed-specific (BS) growth rates. BS growth rates: 60g/ d (Cobb), 46g/ d (CobbSasso), 38g/ d (Sasso) and 30g/ d (Sussex).

**Fig 6:**
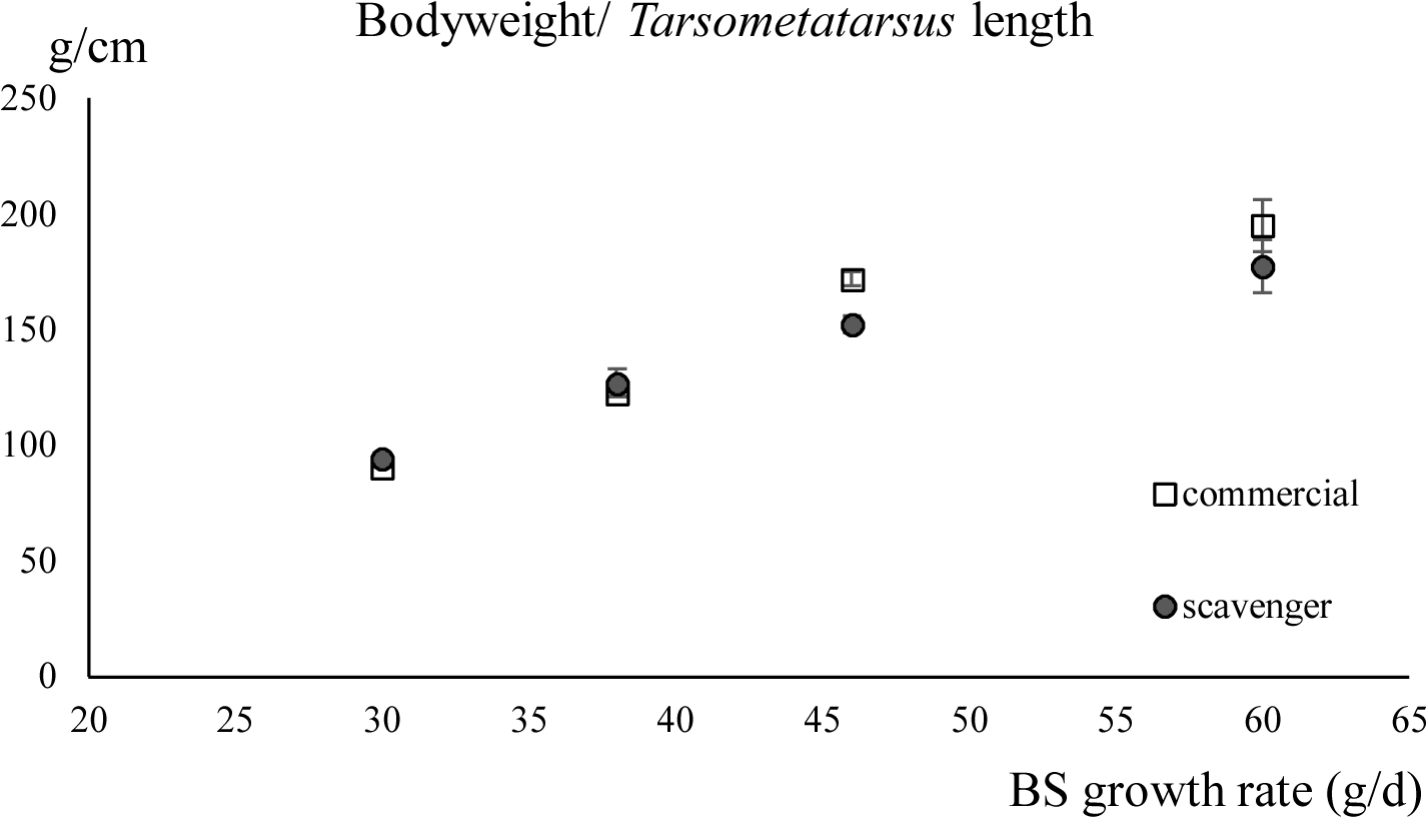
The ratio of bodyweight/ t*arsometatarsus* length (± standard deviation) of chickens fed the scavenger or the commercial diet in function of their breed-specific (BS) growth rates. BS growth rates: 60g/ d (Cobb), 46g/ d (CobbSasso), 38g/ d (Sasso) and 30g/ d (Sussex).

The length of the *tarsometatarsus* increased significantly with increasing breed-specific growth rate (*P* < 0.001) and when fed the commercial diet (*P* < 0.01). No interaction between the factors, diet and breed-specific growth rate, was detected. The results of the linear regression for the ratio of the bodyweight/ *tarsometatarsus* length were determined by the breed-specific growth rate only (*P* < 0.001) Table 2 (Figs 5 and 6).

### Digestibility

The digestibility coefficients of EE and CP were lower on the scavenger diet compared with the commercial diet (*P* < 0.001 and *P* = 0.004 respectively). The EE digestibility of the commercial diet was 92% ± 3% and 82% ± 5% for the scavenger diet. The CP digestibility of the commercial diet was 51% ± 10% and 25% ± 22% for the scavenger diet. There was no significant difference (*P* > 0.05) found between the four breeds according to the digestibility of CF, EE and CP.

### Litter

The litter was observed to be more humid and sticky with increasing breed-specific growth rate and when the birds were fed the scavenger diet (pers. Obs. JP).

## Discussion

All breeds increased their ADFI when fed the scavenger diet. The two slowest growing breeds in this study, Sasso and Sussex, increased their feed intake by 40 and 30% respectively and therefore managed to achieve the same bodyweights as on the commercial diet. The bodyweight of Sussex was even slightly, but not significantly, higher when fed the scavenger diet. The fastest growing breeds, Cobb and CobbSasso, consistently achieved the highest bodyweights compared to the slower growing breeds. Yet, based on the FCR, they should have increased their feed intake by 45 and 40% respectively, in order to achieve the same bodyweights on the scavenger diet as on the commercial diet, which they were not capable of doing. This in contradiction to an experiment by Leeson *et al*. [38] where broilers of a commercial strain increased their feed intake sufficiently as the energy content of the feed decreased. These broilers did manage to maintain their bodyweight over an energy difference of 2.5 MJ. This contrast might be explained by the use of an “older” commercial broiler breed (which was not specified) or by the fact that the energy difference between the diets was lower than in our study, where it was 5.0 MJ/ kg. Olomu and Offiong [39] found no significant effect on either feed intake or weight gain for starting broilers fed diets with an energy difference of 1.7 MJ. This in contrast to the study by Harms *et al*. [40] where a significant effect (*P* < 0.05) of breed, diet and breed × diet interaction was found concerning both weight gain and feed intake. No rating for the growth rates of the different chicken breeds was made in this study. Equally so, Leeson *et al*. [41] noted an increase in feed intake as well as feed intake to bodyweight gain ratio of broilers when the energy and protein level in the feed were diluted. The chickens’ bodyweight was significantly affected on day 42 but not on day 49.

The effects of the scavenger diet on bodyweight seen in our study are in line with Havenstein *et al*. [42] and Grashorn [43]. In the first study, the effect of diet on the bodyweights of two chicken breeds, Ross 308 and Athens-Canadian Randombred, was measured. The diets were an industrial diet from 1957 and one from 2001, the latter one being higher in protein, fat and energy. For the Ross 308 breed a difference in bodyweight of 18.9 and 21.8% for females and males respectively was found and for the Athens-Canadian Randombred breed a difference of 5.4 to 7.8% for females and males respectively was found. The highest weights were always obtained for the 2001 industrial diet, but the differences with the 1957 industrial diet were smaller for the “older” breed. Grashorn [43] found a difference in bodyweight of 8–14% for slow growing broilers, whereas for the fast growing broilers a difference of 25% between diets with a high and low nutrient concentration was seen. Both studies support the association between breed-specific growth rate and the difference in bodyweight when fed a less concentrated diet. None of those two studies monitored the digestibility of nutrients or the feed intake of the chickens.

The stable digestibility of EE and CP throughout time is in line with the results by Batal and Parsons [44]. In that study, an increasing digestibility of EE and CP was found up to the age of 14 and 10 days respectively. Later the digestibility coefficients reached a plateau. These results can be explained by the development of the small intestine, digestive enzymes and villus morphology between hatching and 6 to 14 days of age [45–48]. In addition, the results of Jackson and Diamond [49] and of Proudman *et al*. [50] present equal (both 67%) apparent dry-matter digestibility for both jungle fowls and broilers for similar diets. This in contradiction with Krás *et al*. [51] who found a significant, but “not biologically coherent” effect of age on the digestibility of DM, OM, CP, NDF, ADF and gross energy between the age of 10 and 41 days. The same study also showed a higher ADF-digestibility for the Label Rouge breed compared to the Cobb500 at the age of 31 and 41 days.

In our study, the lower rate of fat and protein digestibility in the scavenger diet compared to the commercial diet could be explained by the high amount of NSP and the lack of added enzymes to break them down. The lack of enzymes enables the fibres to enclose the nutrients and keep them from enzyme break-down with their “cage-effect” [13,52]. The high CF content in the scavenger diet might also state the decrease in manure quality and increase in quantity, due to the water holding capacity of the fibres [53–55]. The higher quantity of excreta in the pens where the birds were fed the scavenger diet might also be caused by the higher feed intake.

Though not significant, the effect of the scavenger diet on the *tarsometatarsus* length was higher with increasing breed-specific growth rate. This length was slightly, but significantly, higher for chickens with a higher breed-specific growth rate (*P* > 0.001) or when fed the commercial diet (*P* < 0.01). The ratio of bodyweight and a skeletal measurement, represented here as the length of the *tarsometatarsus*, can be considered as an indicator for the conformation of the body [26]. The higher this ratio, the more weight per skeletal unit. A slightly, but not significantly, higher ratio of bodyweight/ *tarsometatarsus* length was found for the slow growing breeds on the scavenger diet compared to the commercial diet. This in contrast with Cobb and CobbSasso where this ratio was higher for the commercially fed chickens. The share of muscle, fat and digestive tract in the bodyweight was not determined.

An increasing FCR is generally considered an economic disadvantage but if the scavenger diet can be obtained at much lower cost than the commercial diets, this perspective might change. For example, in a rural situation, where a scavenger diet can (partially) be found in the environment and is available at libitum, our results suggest that scavenging chickens might achieve the same bodyweights as when they were fed a commercial diet. Still, factors such as disease, water availability and housing must be controlled [56,57]. In addition, chickens that are able to find (a share of) their feed in nature will minimize the competition between humans and livestock for feed/food resources. However, the results of this experiment also indicate that industrial broiler breeds will not be able to increase their feed intake sufficiently in order to achieve the same bodyweight on a diet with lower energy and nutrient density (such as the scavenger diet in the present study) as they would do on a current commercial diet.

## Conclusion

The advantage of selecting fast growing broiler chickens on concentrated diets decreases rapidly when less concentrated diets are given. This urges us to reconsider the current selection criteria when considering increasing the amounts of by-products in poultry feed as driven by the feed-food competition. For slow growing chicken breeds—such as the ones used by smallholders in developing countries—the added value of using a concentrated diet versus a typical scavenger diet is low. These differences in growth performance are caused more commonly by differences in feed intake than by differences in digestibility.

## Supporting Information

S1 Data Dataset per pen per week. (XLSX)
